# Risk factors for post‑retrograde cholangiopancreatography pancreatitis in patients with common bile duct stones: A meta‑analysis

**DOI:** 10.3892/etm.2023.12320

**Published:** 2023-11-24

**Authors:** Bo Zhou, Liyuan Zhao, Xinfeng Xing, Hai Wang, Asihati Kuwantai, Kai Chen

**Affiliations:** 1 Department of Hepatobiliary Surgery, The Fifth Affiliated Hospital of Xinjiang Medical University, Urumqi, Xinjiang Uygur Autonomous Region 830011, P.R. China; 2 Department of Gynecology, The First Affiliated Hospital of Xinjiang Medical University, Urumqi, Xinjiang Uygur Autonomous Region 830054, P.R. China

**Keywords:** common bile duct stones, endoscopic retrograde cholangiopancreatography, pancreatitis, risk factors, meta-analysis

## Abstract

Endoscopic retrograde cholangiopancreatography (ERCP) has become a common treatment method for common bile duct stones. However, ERCP is also associated with a high risk of post-endoscopic retrograde cholangiopancreatography pancreatitis (PEP). Identification of risk factors is essential for reducing the incidence of PEP. The present study aimed to summarize the risk factors for PEP by performing a meta-analysis. Therefore, studies published between 2000 and 2022 were screened in PubMed, Cochrane Library, Embase, Web of Science, China National Knowledge Infrastructure, Wanfang Digital Periodicals and the Weipu Database, with no language restrictions. Newcastle-Ottawa Scale was used to assess the quality of the included studies. Stata 17.0 software was utilized for the meta-analysis of 14 possible risk factors. Overall, 15 high-quality studies were included into the present meta-analysis. The results showed that female [odds ratio (OR), 1.42; 95% CI, 1.23-1.64), age <60 years (OR, 1.53; 95% CI, 1.06-2.21), difficult intubation (OR, 4.87; 95% CI, 2.73-8.68), ≥3 cannulation attempts (OR, 9.64; 95% CI, 4.16-22.35), cannulation time ≥10 min (OR, 2.37; 95% CI, 1.67-3.35), history of pancreatitis (OR, 2.95; 95% CI, 1.06-5.51), pancreatic duct visualization (OR, 3.63; 95% CI, 2.47-5.34) and sphincter of Oddi dysfunction (OR, 5.72; 95% CI, 1.80-18.24) are potential risk factors for PEP (P<0.05). In conclusion, the present meta-analysis suggests that PEP can be affected by several risk factors, particularly the technique-related factors such as the frequency and time of cannulation. Therefore, effective precautions should be taken as early as possible to reduce the incidence of PEP.

## Introduction

Common bile duct stones (CBDS) is a common biliary tract disease that is caused by biliary tract infection, cholestasis, biliary mechanical changes, hepatitis B virus and parasites, occurring in 10-15% of all patients with cholelithiasis (1). The incidence of cholelithiasis has been increasing annually, with a prevalence of 10% in American adults, and 5.9-21.9 and 3.2-15.6% in Western Europe and Asia, respectively (2). CBDS is characterized by complex and variable clinical manifestations, with dispensable symptoms. Symptomatic CBDS patients often exhibit Charcot's triad, which includes epigastric pain, jaundice, as well as chills and fever, and/or Reynolds pentad, consisting of Charcot's triad with hypotensive shock and neuropsychiatric symptoms, which commonly appear during the attack period of this disease (1). Asymptomatic patients do not present with these clinical features as they have small BDSs that are not enough to cause biliary obstruction. Generally, endoscopic resection is recommended after the diagnosis of CBDS regardless of the presence or absence of symptoms, as asymptomatic CBDS have a potential risk of causing obstructive jaundice, acute cholangitis and biliary pancreatitis (3,4). Currently, endoscopic retrograde cholangiopancreatography (ERCP) is widely considered as an effective treatment approach for CBDS. However, it is also a high-risk procedure accompanied due to the risk of several complications, such as post-ERCP pancreatitis (PEP), short- or long-term cholecystitis and recurrence of CBDS (5), which can be fatal in severe cases (6). The incidence of PEP is estimated to be 1-40% (7), but understanding into its pathogenesis and risk factors remains limited.

Identification of risk factors associated with the development of PEP could contribute to its prevention in clinical practice. For high-risk patients, ERCP should be avoided, with whom protective endoscopic intervention should instead be performed (8). In addition, the identification of risk factors for PEP may contribute to the reduction of treatment costs whilst increasing clinical benefits.

Therefore, in the present study, a meta-analysis was performed to screen risk factors for PEP, with the ultimate aim of reducing its incidence whilst improving prognosis.

## Materials and methods

### Search strategies

Relevant studies were screened in PubMed (https://pubmed.ncbi.nlm.nih.gov/), Cochrane Library (https://www.cochrane.org/zh-hans/welcome), Embase (https://www.embase.com/landing?status=grey), Web of Science (https://www.webofscience.com/wos/alldb/basic-search), China National Knowledge Infrastructure (CNKI) (https://www.cnki.net/), Wanfang Digital Periodicals (WANFANG) (https://www.wanfangdata.com.cn/index.html) and the Weipu Database (VIP) (http://www.cqvip.com/). The search criteria were studies published between 2000 and 2022, with no language restrictions. The search strategy was determined using several combinations of subject words and free-text words. The key words used in the literature search were as follows: ‘Choledocholithiasis’ OR ‘Cholelithiasis, Common Bile Duct’, AND ‘ERCP’ OR ‘Retrograde Cholangiopancreatography, Endoscopic’ OR ‘Cholangiopancreatographies, Endoscopic Retrograde’ OR ‘Endoscopic Retrograde Cholangiopancreatographies’ OR ‘Retrograde Cholangiopancreatographies, Endoscopic’ OR ‘Endoscopic Retrograde Cholangiopancreatography’, AND ‘pancreatitis’ OR ‘Pancreatitis, Acute Edematous’ OR ‘Acute Edematous Pancreatitides’ OR ‘Edematous Pancreatitides, Acute’ OR ‘Edematous Pancreatitis, Acute’ OR ‘Pancreatitides, Acute Edematous’ OR ‘Acute Edematous Pancreatitis’ OR ‘Pancreatic Parenchymal Edema’ OR ‘Edema, Pancreatic Parenchymal’ OR ‘Pancreatic Parenchymal Edemas’ OR ‘Parenchymal Edema, Pancreatic’ OR ‘Pancreatic Parenchyma with Edema’ OR ‘Pancreatitis, Acute’ OR ‘Acute Pancreatitis’ OR ‘Acute Pancreatitides’ OR ‘Pancreatitides, Acute’ OR ‘Peripancreatic Fat Necrosis’ OR ‘Fat Necrosis, Peripancreatic’ OR ‘Necrosis, Peripancreatic Fat’ OR ‘Peripancreatic Fat Necroses’.

### Inclusion and exclusion criteria

The inclusion criteria were as follows: i) Involvement of patients with CBDS who underwent ERCP for removal of stones, including cholesterol stones, pigment stones and mixed stones; ii) Univariate or multivariate analysis results of the risk factors for PEP, including relative risk (RR), hazard ratio (HR) or odds ratio (OR) and corresponding 95% CI, being available; iii) diagnosis of PEP based on increased serum amylase levels (>3-folds at 24 h after ERCP), accompanied by obvious abdominal pain or imaging data suggesting PEP; iv) studies including >90 cases for risk evaluation and providing sufficient data to meet the analysis requirements of the statistical software; and iv) studies with available full-text articles. In addition, if the same group of authors published multiple studies using patients from the same hospital over a number of years, then only the most recent study would be included whilst discarding others. Review articles or case reports, studies with repeated results and/or abnormal data that could not be extracted or utilized, and those with unknown data descriptions or inaccurate definitions were excluded from the present meta-analysis.

### Data extraction and quality evaluation

Literature screening was independently performed by two investigators according to the inclusion and exclusion criteria. Subsequently, both investigators reviewed the full texts of the screened studies to extract the key data, including author, publication year, country, study type, participant characteristics, sample size, risk factors, RR/HR/OR, 95% CI and confounding factor adjustment. Quality evaluation of the studies was performed independently by three investigators. In cases of disputes among the investigators, a comprehensive discussion would be held together with an experienced investigator (KC), until a consensus was reached. Newcastle-Ottawa Scale (NOS) is frequently used to evaluate the quality of non-randomized studies in meta-analyses (9) and to assess the methodological quality of the observational studies. Evaluation items in the scale included participant selection (score, 4 points), comparability between groups (score, 2 points) and exposure status (score, 3 points). Finally, only high-quality studies with a NOS score of >6 were included in the meta-analysis. Patients who developed PEP after ERCP were allocated into the treatment group, whilst those who did not develop PEP were included in the control group.

### Statistical analysis

Data analysis was performed using the Stata software (version 17.0; StataCorp LP). The effect indicator of the enumeration data was OR, whereas that of the measurement data was the mean difference (MD). The I^2^ test was utilized to evaluate the heterogeneity of the included studies. The random-effects model was utilized for meta-analysis regardless of the I^2^ value. Sensitivity analysis was performed by comparing the result consistency (I^2^ value) between the fixed-effects and random-effects models and eliminating studies with greater impact (i.e., effect sizes of studies exceeded the upper limit of the CI value) on the consistency of the combined results. A funnel plot and Egger's test were used to assess publication bias in the included studies. P<0.05 was considered to indicate a statistically significant difference.

## Results

### Basic characteristics of the included studies

**Figure 1:**
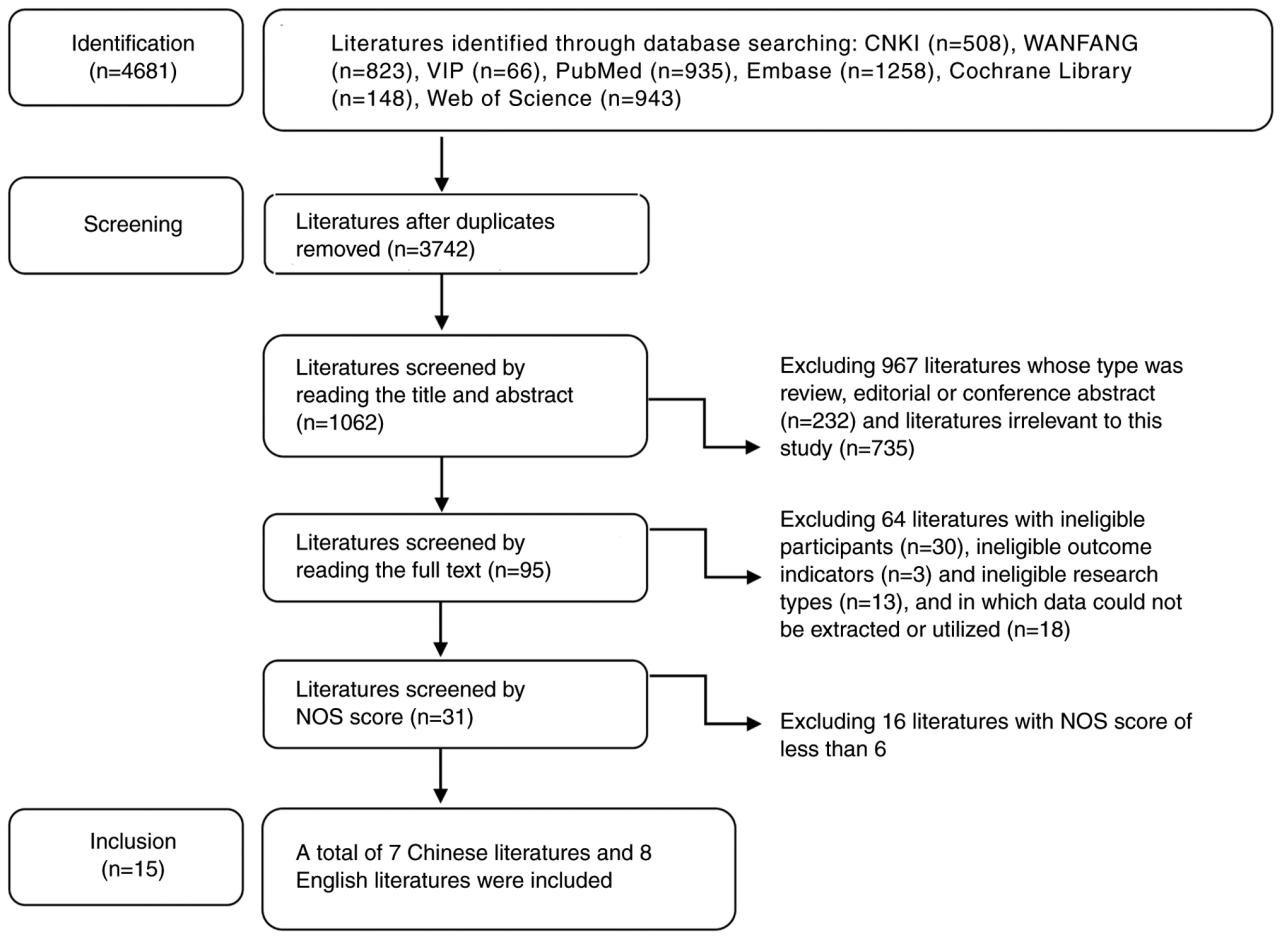
Flow chart of literature screening. CNKI, China National Knowledge Infrastructure; VIP, Weipu Database; WANFANG, Wanfang Digital Periodicals; NOS, Newcastle-Ottawa.

A total of 4,681 studies were initially retrieved and 1,062 remained after removing all duplicate publications and the initial screening by title and abstract. Subsequently, after excluding all review articles, editorial or conference abstracts and irrelevant studies, 95 studies remained. Thereafter, 64 studies were then excluded due to ineligible participants (n=30), outcome indicators (n=3), research types (n=13) and failure in data extraction and utilization (n=18). Finally, after excluding 16 studies with NOS score <6, 15 high-quality studies, including seven in Chinese and eight in English, were included into the present meta-analysis (Fig. 1). The basic characteristics of the included studies are listed in Table I (10-24). These studies were from China, Sweden, Japan, Korea, Greece, the USA and Germany, including case-control, prospective cohort and retrospective cohort studies. The smallest sample size was 92 participants (10) and the largest was 12,718(16).

### Meta-analysis

The meta-analysis of the potential risk factors for the onset of PEP is shown in Table II. The risk factors showing a significant effect included age <60 years, female sex, difficult cannulation, ≥3 cannulation attempts, cannulation time ≥10 min, pancreatitis history, pancreatic duct visualization and sphincter of Oddi dysfunction (SOD) (P<0.05). Among them, difficult cannulation was directly described in the articles, without being clearly defined. Therefore, the two major factors generally associated with difficult cannulation, namely ≥3 cannulation attempts and cannulation time of ≥10 min, were considered to be independent risk factors.

### Age <60 years

**Figure 2:**
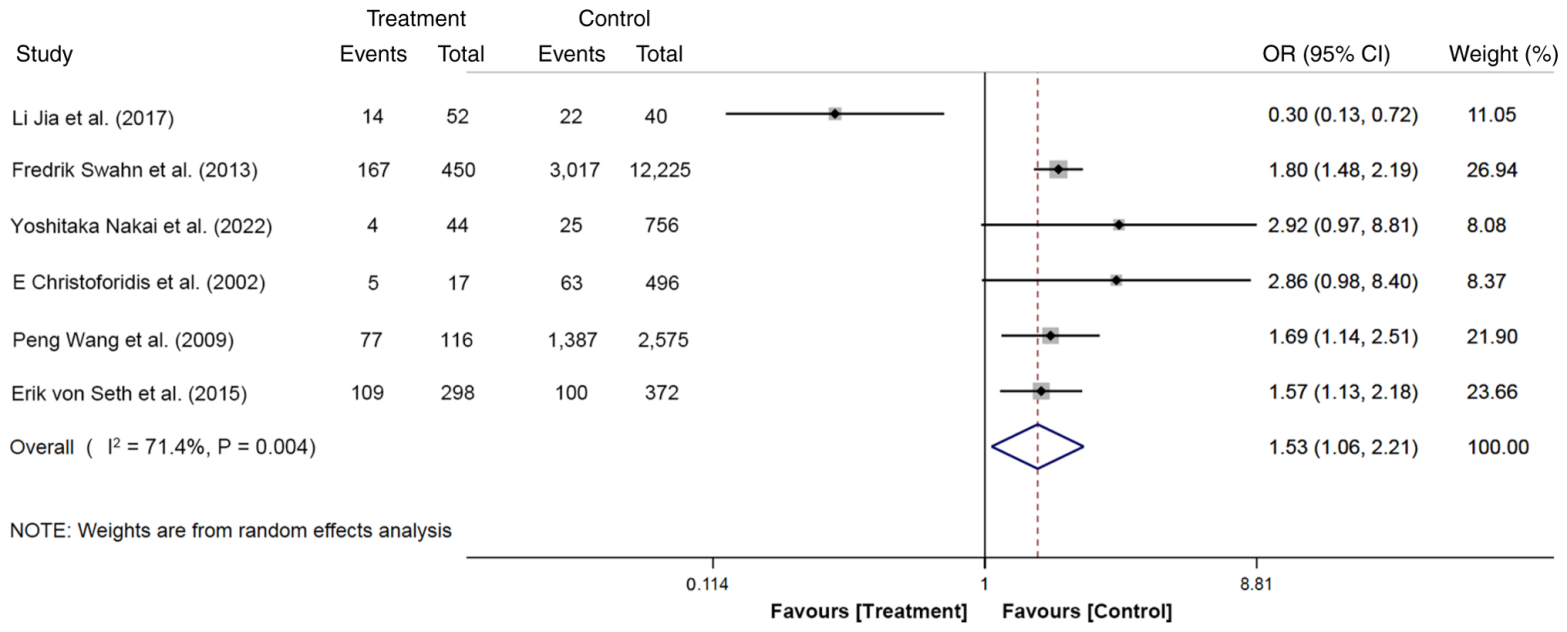
Forest plot of age <60 years using random-effects model based on patients with PEP (treatment group) vs. patients with no PEP (control group). The ‘Events’ column represents the number of patients of age <60 years. The ‘Total’ column represents the total number of patients. OR, odd ratio; PEP, post-endoscopic retrograde cholangiopancreatography pancreatitis.

The effect of age on PEP was reported in six studies, with a total of 17,441 participants. Among them, 977 cases (age <60 years, 376 cases; age >60 years, 601 cases) were included in the treatment group and 16,464 cases (age <60 years, 4,614 cases; age >60 years, 11,850 cases) in the control group. The random effects model was used for the meta-analysis. The results showed that the risk of PEP in patients aged <60 years was 1.53-fold higher compared with those aged >60 years old (OR, 1.53; 95% CI, 1.06-2.21; P<0.05; Fig. 2). These findings suggest that younger patients with CBDS could be at higher risk of developing PEP.

### Female patients

**Figure 3:**
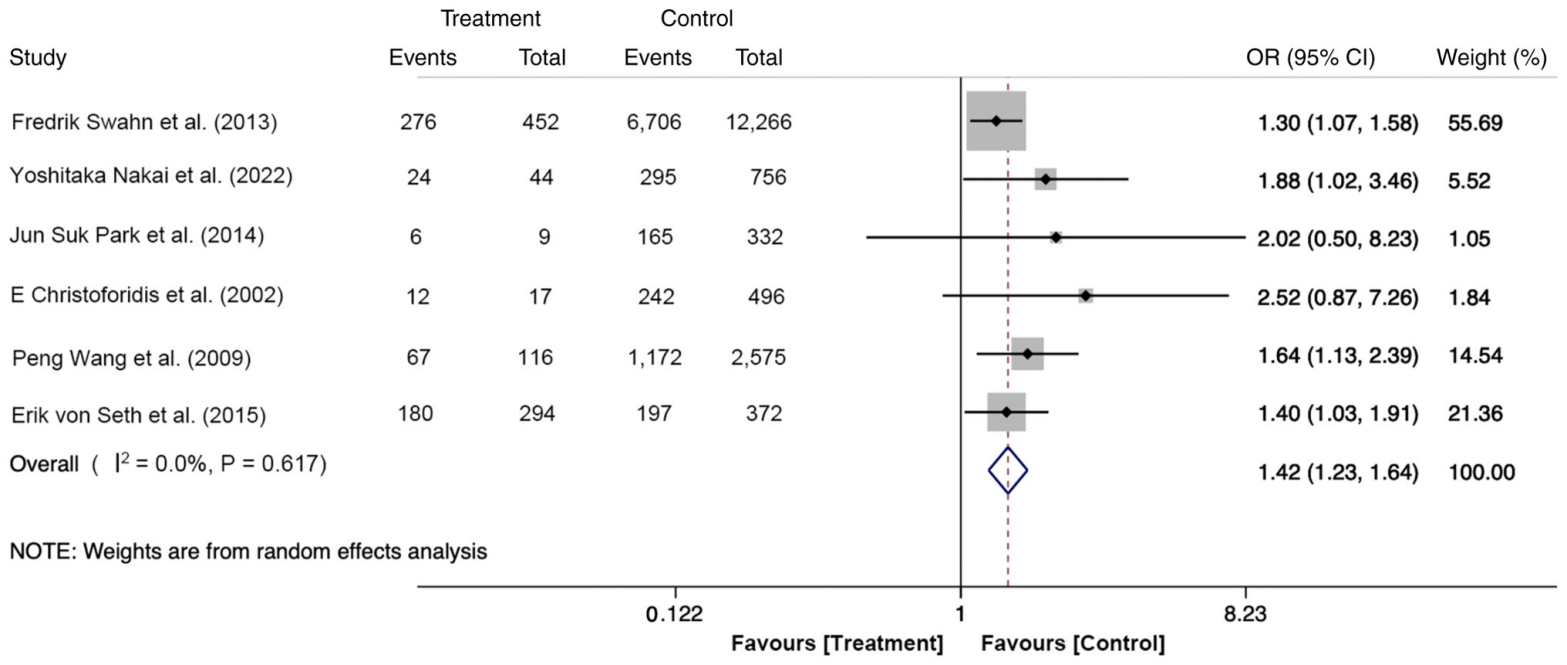
Forest plot of female using random-effects model based on patients with PEP (treatment group) vs. patients with no PEP (control group). The ‘Events’ column represents the number of female patients. The ‘Total’ column represents the total number of patients. OR, odd ratio; PEP, post-endoscopic retrograde cholangiopancreatography pancreatitis.

The effect of sex on PEP was reported in six studies, including 17,729 participants, with 932 cases in the treatment group (males, 367 cases; females, 565 cases) and 16,797 cases in the control group (males, 8,021 cases; females, 8,776 cases). The fixed-effects model was used for this meta-analysis. The results indicated that the risk of PEP occurrence in female patients was 1.42-fold higher compared with male counterparts (OR, 1.42; 95% CI, 1.23-1.64; P<0.05; Fig. 3). Taken together, young female patients could be considered a high-risk group for PEP.

### Difficult cannulation

**Figure 4:**
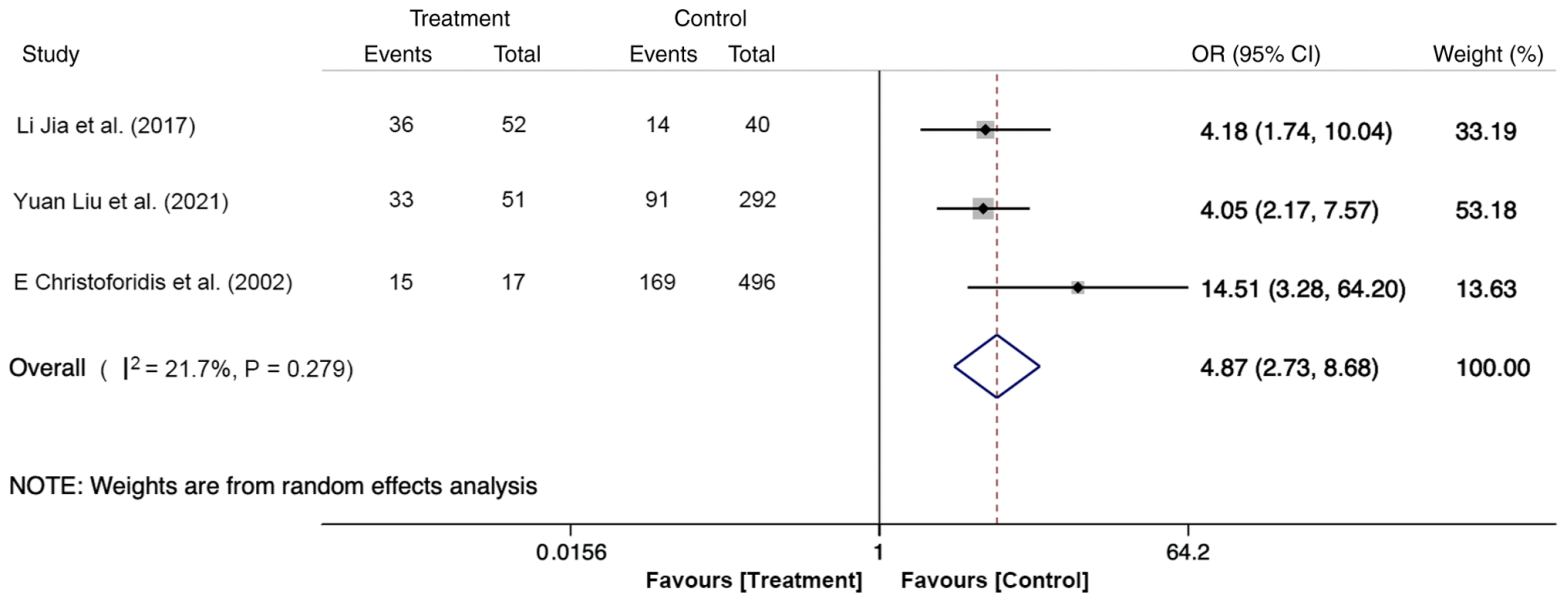
Forest plot of difficult cannulation using random-effects model based on patients with PEP (treatment group) vs. patients with no PEP (control group). The ‘Events’ column represents the number of patients showing difficult cannulation. The ‘Total’ column represents the total number of patients. OR, odd ratio; PEP, post-endoscopic retrograde cholangiopancreatography pancreatitis.

There were three studies describing the effect of difficult cannulation on PEP. A total of 948 participants were included in the meta-analysis, with 120 cases (difficult cannulation, 84 cases; easy cannulation, 36 cases) in the treatment group and 828 cases (difficult cannulation, 274 cases; easy cannulation, 554 cases) in the control group. The meta-analysis was performed utilizing the fixed-effects model and the results demonstrated that the risk of PEP in patients with difficult cannulation was 4.97-fold higher compared with those with easy cannulation (OR, 4.87; 95% CI, 2.73-8.68; P<0.05; Fig. 4). These findings suggest that difficulty in cannulation is associated with the surgical operation. Therefore, attention should be paid to the technical clinical factors to reduce the incidence of PEP.

### Cannulation attempts ≥3

**Figure 5:**
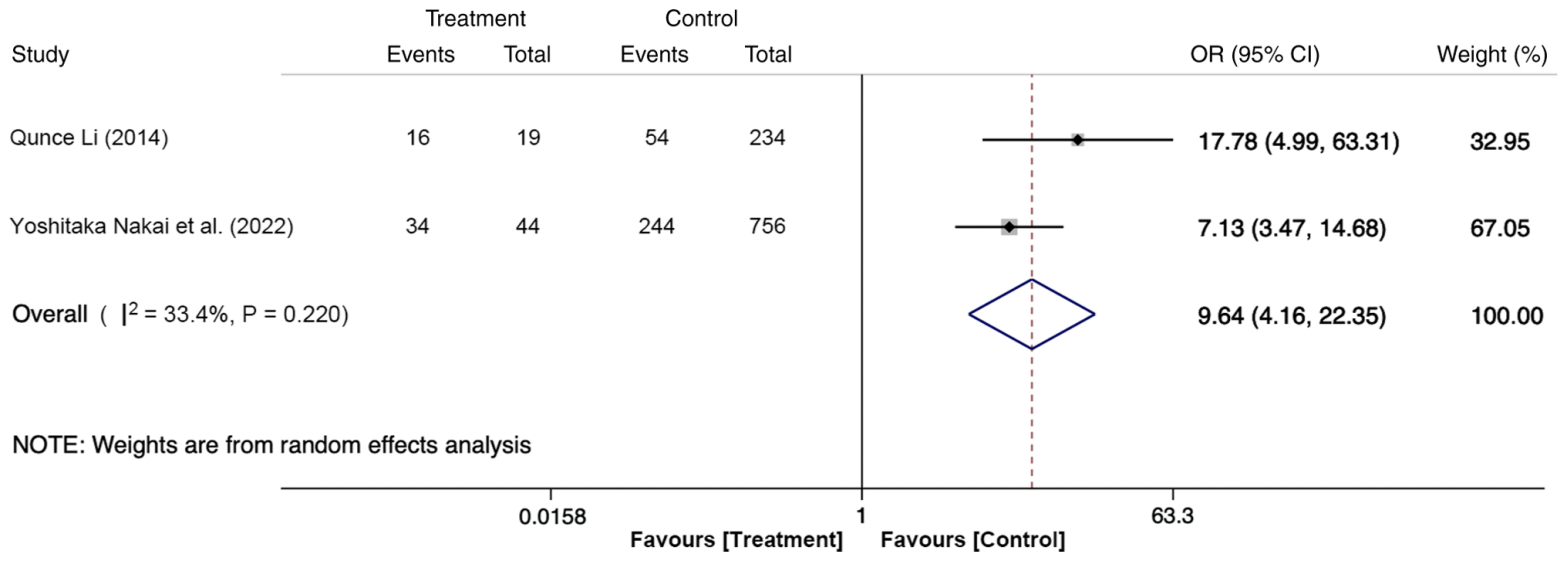
Forest plot of ≥3 cannulation attempts using random-effects model based on patients with PEP (treatment group) vs. patients with no PEP (control group). The ‘Events’ column represents the number of patients with ≥3 cannulation attempts. The ‘Total’ column represents the total number of patients. OR, odd ratio; PEP, post-endoscopic retrograde cholangiopancreatography pancreatitis.

A total of two articles, including 1,053 participants, with 63 (≥3 cannulation attempts, 50 cases; <3 cannulation attempts, 13 cases) in the treatment group and 990 (≥3 cannulation attempts, 298 cases; <3 cannulation attempts, 692 cases) in the control group, reported the effect of the number of cannulation attempts on PEP. The fixed-effects model revealed that the risk of PEP in participants with ≥3 cannulation attempts was 8.98-fold higher compared with that with <3 cannulation attempts (OR, 9.64; 95% CI, 4.16-22.35; P<0.05; Fig. 5). This result suggests that the occurrence of PEP is positively associated with the number of cannulation attempts.

### Cannulation time ≥10 min

**Figure 6:**
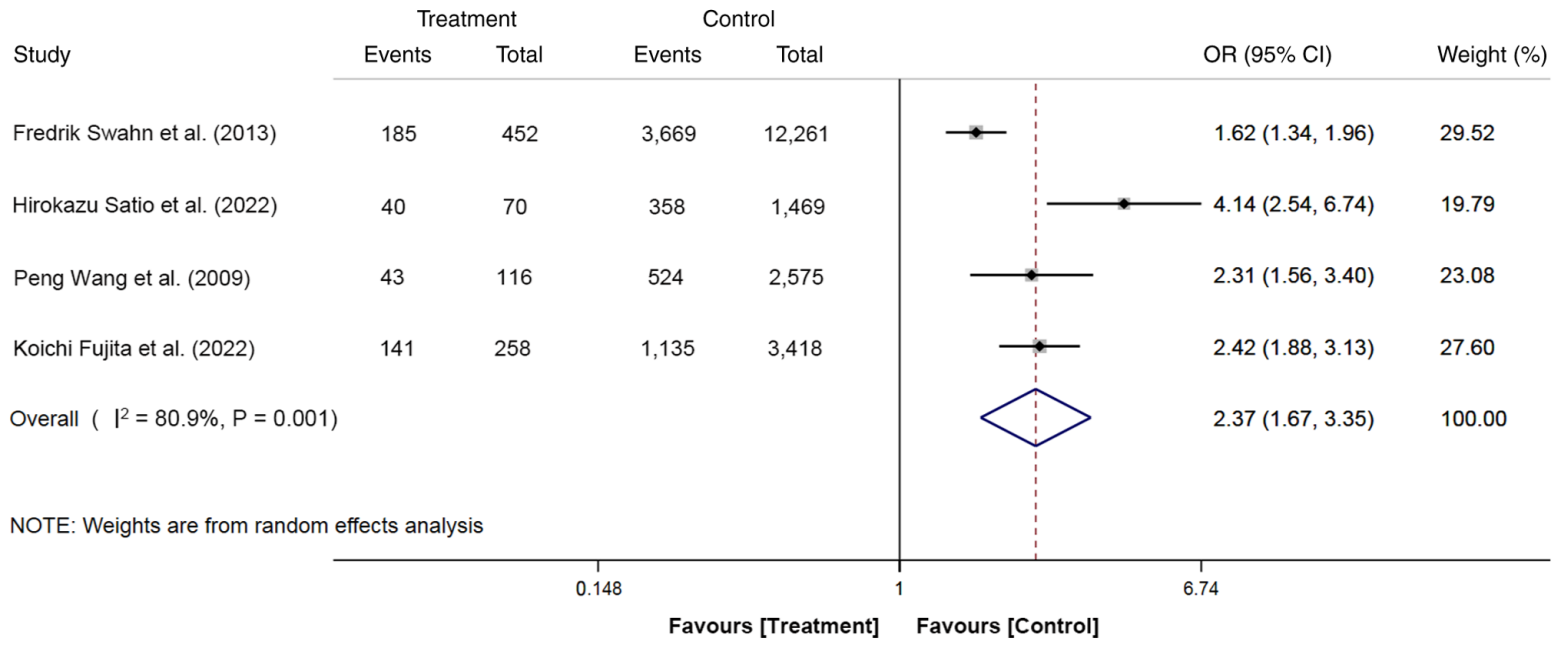
Forest plot of cannulation time ≥10 min using random-effects model based on patients with PEP (treatment group) vs. patients with no PEP (control group). The ‘Events’ column means the number of patients with a cannulation time ≥10 min. The ‘Total’ column represents the total number of patients. OR, odd ratio; PEP, post-endoscopic retrograde cholangiopancreatography pancreatitis.

The effects of cannulation time on PEP were reported in four studies. A total of 20,619 participants were included, with 896 cases (cannulation time ≥10 min, 409 cases; cannulation time <10 min, 487 cases) in the treatment group and 19,723 cases (cannulation time ≥10 min, 5,686 cases; cannulation time <10 min, 14,037 cases) in the control group. The meta-analysis was performed using the random-effects model and the results demonstrated that the risk of PEP in patients with cannulation time ≥10 min was 2.37-fold higher compared with those with a cannulation time <10 min (OR, 2.37; 95% CI, 1.67-3.35; P<0.05; Fig. 6). This finding suggested that cannulation time of ≥10 min was a risk factor for PEP.

### Previous history of pancreatitis

**Figure 7:**
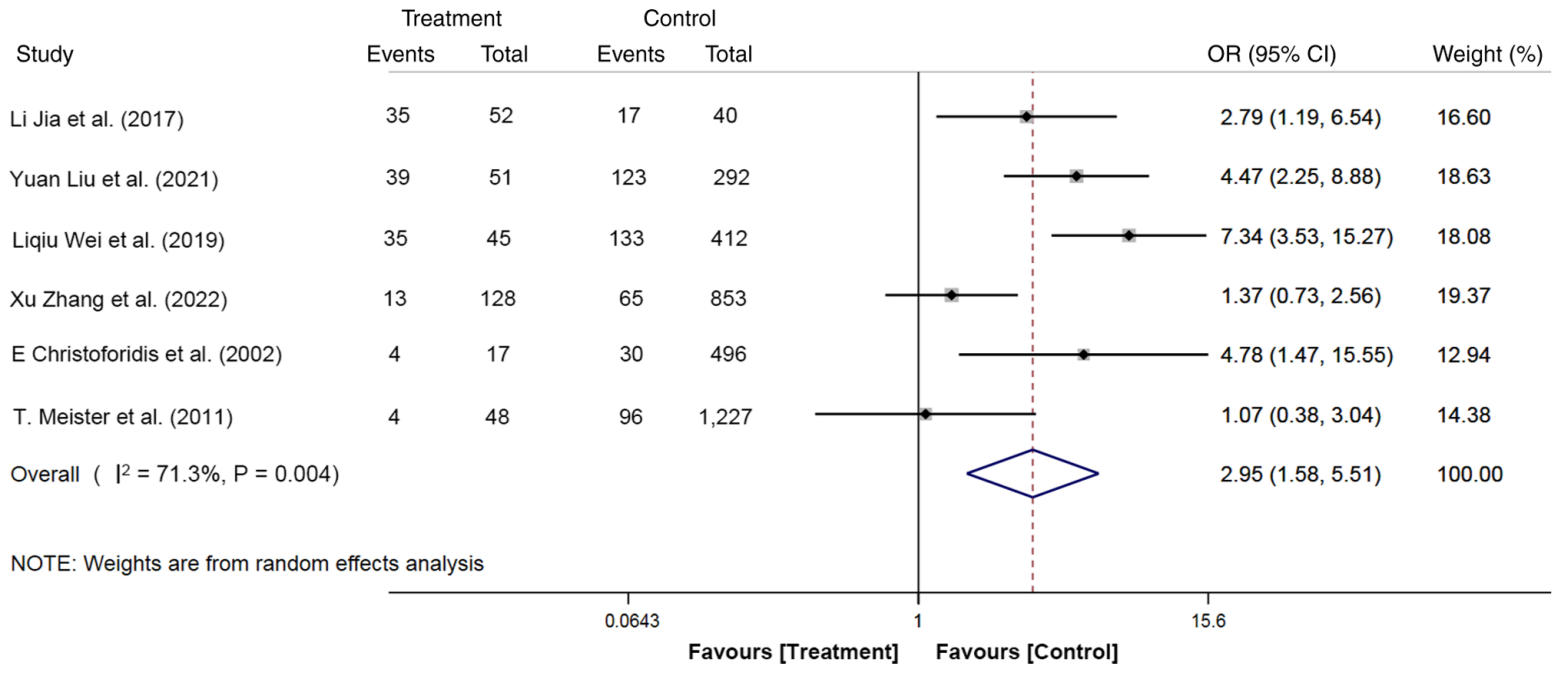
Forest plot of a history of pancreatitis using random-effects model based on patients with PEP (treatment group) vs. patients with no PEP (control group). The ‘Events’ column represents the number of patients with a history of pancreatitis. The ‘Total’ column represents the total number of patients. OR, odd ratio; PEP, post-endoscopic retrograde cholangiopancreatography pancreatitis.

A total of six articles with 3,661 participants reported the effects of previous pancreatitis history on PEP, including 341 cases (pancreatitis history, 130 cases; without pancreatitis history, 211 cases) in the treatment group and 3,320 cases (pancreatitis history, 464 cases; without pancreatitis history, 2,856 cases) in the control group. Based on the random-effects model, patients with a history of pancreatitis showed a 2.95-fold increased risk of developing PEP compared with those with no previous pancreatitis history (OR, 2.95; 95% CI, 1.58-5.51; P<0.05; Fig. 7). Overall, this finding suggests that patients with a history of pancreatitis exhibit a higher risk of PEP recurrence, highlighting that great attention should be paid to the history of pancreatitis.

### Pancreatic duct visualization

**Figure 8:**
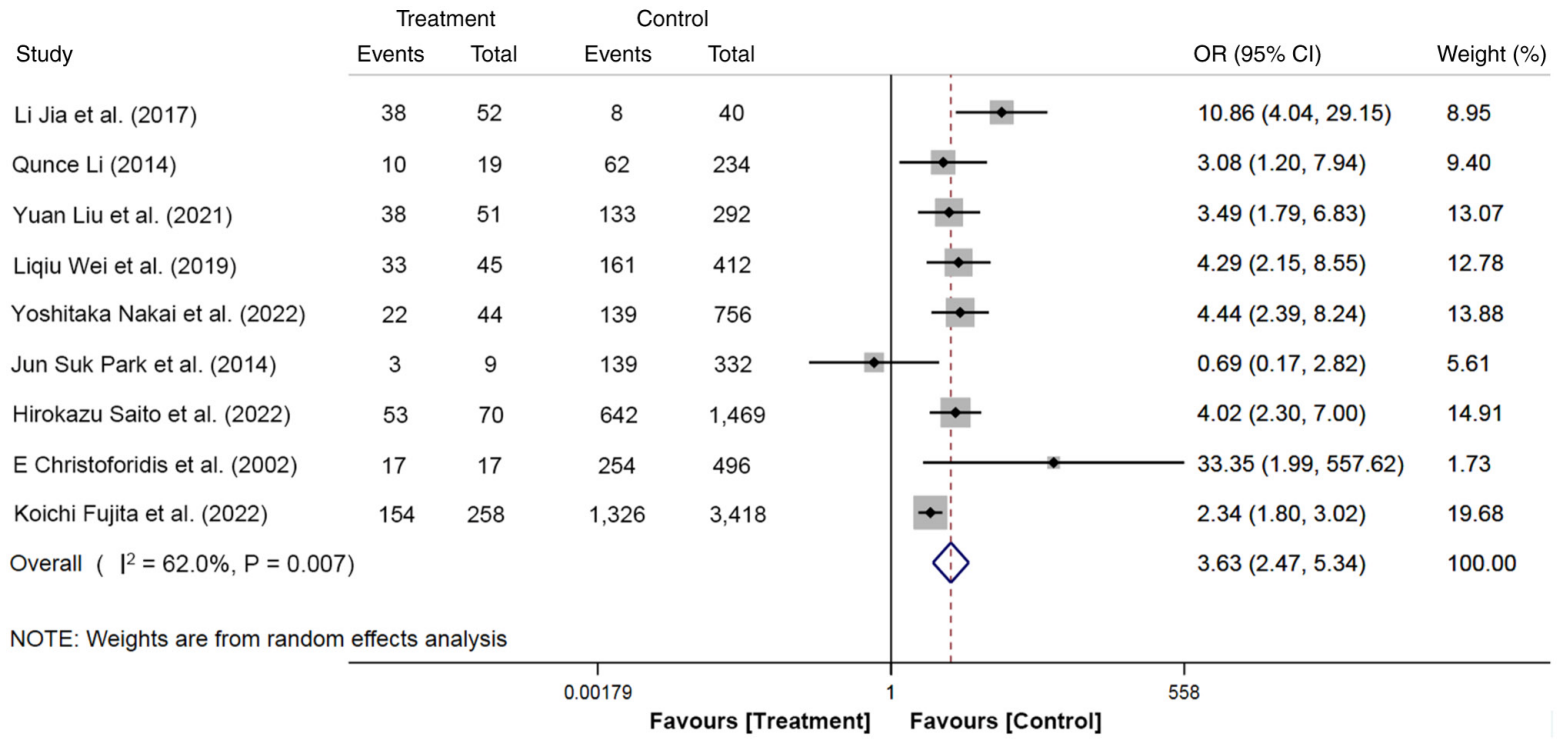
Forest plot of pancreatic duct visualization using random-effects model based on patients with PEP (treatment group) vs. patients with no PEP (control group). The ‘Events’ column represents the number of patients with pancreatic duct visualization. The ‘Total’ column represents the total number of patients. OR, odd ratio; PEP, post-endoscopic retrograde cholangiopancreatography pancreatitis.

The effect of pancreatic duct visualization on the development of PEP was reported in nine studies, involving 8,014 participants, with 565 cases (pancreatic duct visualization, 368 cases; without pancreatic duct visualization, 197 cases) in the treatment group and 7,449 cases (pancreatic duct visualization, 2,864 cases; without pancreatic duct visualization, 4,585 cases) in the control group. The results of the random effect model illustrated that patients with pancreatic duct visualization had a 3.63-fold increased risk of developing PEP compared with those without pancreatic duct visualization (OR, 3.63; 95% CI, 2.47-5.34; P<0.05; Fig. 8). These results suggest that perioperative pancreatic duct visualization may present a potential risk factor for PEP.

### SOD

**Figure 9:**
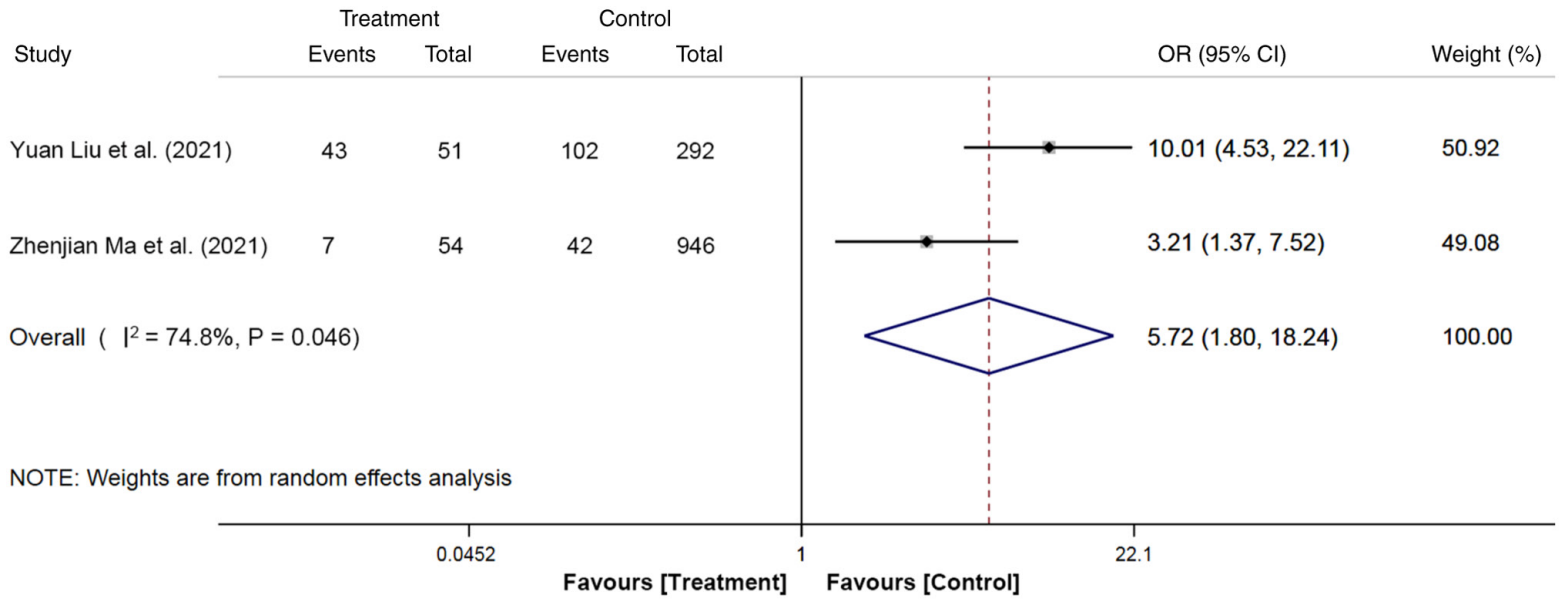
Forest plot of sphincter of Oddi dysfunction using random-effects model based on patients with PEP (treatment group) vs. patients with no PEP (control group). The ‘Events’ column represents the number of patients with sphincter of Oddi dysfunction. The ‘Total’ column represents the total number of patients. OR, odd ratio; PEP, post-endoscopic retrograde cholangiopancreatography pancreatitis.

The association between SOD and the risk of PEP was reported in two studies, with a total of 1,343 participants, including 105 cases (SOD, 50 cases; no SOD, 55 cases) in the treatment group and 1,238 participants (SOD, 144 cases; no SOD, 1,094 cases) in the control group. The random-effects model demonstrated that patients with SOD exhibited a 5.72-fold higher risk of developing PEP compared with those without SOD (OR, 5.72; 95% CI, 1.80-18.24; P<0.05; Fig. 9).

### Publication bias

**Figure 10:**
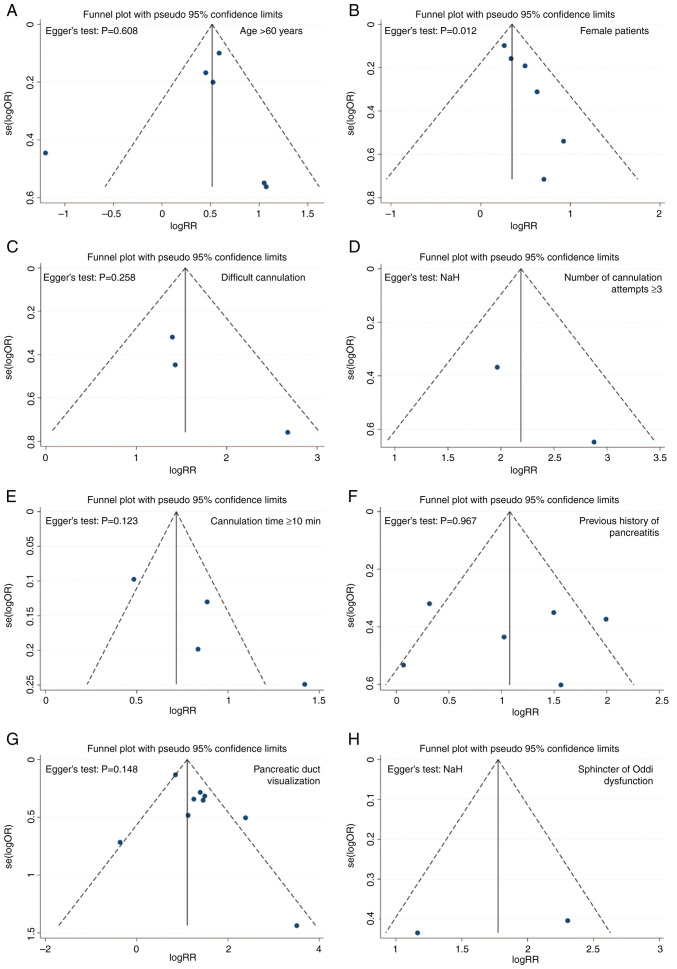
Funnel plots and results of Egger's test used for evaluating publication bias of the included studies. (A) Age >60 years. (B) Female patients. (C) Difficult cannulation. (D) Number of cannulation attempts ≥3. (E) Cannulation time ≥10 min. (F) Previous history of pancreatitis. (G) Pancreatic duct visualization. (H) Sphincter of Oddi dysfunction. NaN, the P-value in Egger's test could not be obtained; OR, odds ratio; RR, relative risk.

The funnel plot and Egger's test were used to assess publication bias. The analysis suggested that there was no evident publication bias (Egger's test, P>0.05), except for bias in the risk factor of female patients (P<0.05; Fig. 10). The bias in the female risk factor may be due to the small quantity of the included studies.

### Sensitivity analysis

The sensitivity of risk factors was evaluated using the fixed-effects and random-effects models. The results revealed a good consistency of the risk factors, except for cannulation time ≥10 min, supporting the stability of the results. The instability that existed in cannulation time ≥10 min may be attributed to differences in publication countries and study types among the included studies.

## Discussion

CBDS is a common condition that is typically not accompanied with any symptoms in particular (25). However, 10-25% all patients with CBDS may experience biliary colic, obstructive jaundice, pancreatitis, obstructive suppurative cholangitis or even mortality. Among them, 1-2% patients may develop serious complications (26). A recent study showed that the incidence of CBDS was significantly higher in male and female patients who are >60 and >50 years of age, respectively, compared with that in the younger population (27). CBDS can be induced by several factors, including bile hyposecretion, as a result of structural malformations of the biliary tract (28). Currently, ERCP is considered to be the most effective treatment approach for CBDS-induced obstructive jaundice (29). Saito *et al* (30) previously investigated the feasibility and safety of single-stage endoscopic stone removal in patients with CBDS. It was found that single-stage endoscopic stone removal could not increase the incidence of ERCP-related complications, even instead significantly reducing hospital stay (30). Therefore, patients with CBDS may benefit from ERCP.

Over the past decade, minimally invasive surgery for CBDS has been gradually replacing traditional laparotomy techniques (31). The most common surgical method for treating gallstones combined with CBDS is one-stage ERCP stone removal, followed by two-stage laparoscopic cholecystectomy (32). For ERCP, a long tube with a camera (endoscope) is typically inserted through the mouth, esophagus and stomach into the duodenum, which is then moved retrogradely along the sphincter of Oddi to remove the stones (33). However, although the risk of surgery is reduced, the pain of multiple operations, increased hospital stay and excessive cost cannot be avoided. Additionally, the implementation of endoscopic sphincterotomy and endoscopic papillary balloon dilation in ERCP may lead to dysfunction of the nipple sphincter of Oddi, which can result in postoperative complications, such as abdominal pain, fever, jaundice, biliary tract infections and even acute pancreatitis in severe cases (34,35). Therefore, efforts have been made to prevent PEP and reduce its severity.

Several patient-, technique- and operator-related factors were previously found to be associated with the risk of PEP development (36). Patient-related factors commonly include age, sex, a previous history of pancreatitis and SOD. By contrast, technique-related factors mainly include difficult nipple cannulation, nipple sphincterotomy, pancreatic duct sphincterotomy, contrast medium injection in pancreatic duct and bile duct balloon dilatation. In addition, improper training, inexperience and participation of advanced students are considered as one of the most common operator-related factors associated with the onset of PEP. In the present study, 15 high-quality observational studies were rigorously assessed, which reported data on risk factors for PEP. Consistent with previous studies, the present meta-analysis verified that age <60 years, female sex, history of pancreatitis, SOD, difficult cannulation, ≥3 cannulation attempts, cannulation time ≥10 min and pancreatic duct visualization are among the risk factors for PEP (21,37).

The mechanism underlying PEP remains unclear. Results of the present meta-analysis revealed that patients aged <60 years were at high risk of developing PEP. It was previously reported that pancreatic exocrine function becomes progressively weaker with increasing age, which may account for the reduced incidence of PEP in older patients (38). Additionally, compared with male individuals, female patients are more prone to biliary stones due to their higher blood lipid levels, who also tend to be more likely to undergo ERCP to detect suspected biliary stones or SOD (7). This finding may explain the higher incidence of PEP in the female population. In a previous study, the risk of developing PEP in patients with SOD was found to be twice as high compared with that in normal individuals (39). This could be caused by the functional restriction of the biliopancreatic channel, Oddi sphincter diastolic dysfunction and pancreatic juice reflux in patients with SOD. Furthermore, a previous study demonstrated that a previous history of pancreatitis and PEP increased the incidence of PEP by 2.03- and 2.9-fold, respectively (40). This finding could be due to biliary dysfunction observed in patients with a history of pancreatitis. The aforementioned two factors accompanied by SOD could further increase the risk of PEP associated with ERCP.

The present results also showed that the risk of PEP in patients with difficult cannulation was 4.97-fold higher compared with that in patients with easy cannulation. Difficult cannulation can be associated with several factors, such as the condition of patients and more specifically with the experience and skills of the operators. A previous study showed that difficult biliary cannulation was often associated with a smaller sized nipple hole (41). Furthermore, since the bile duct pressure is low in the absence of cholestasis, capillary orifices can be smaller in asymptomatic patients with CBDS compared with symptomatic ones, and the smaller capillary orifices may promote PEP (42). Intubation time ≥10 min or ≥3 intubation attempts are associated with difficult intubation during clinical practice (43). In the present meta-analysis, these two parameters were identified to be risk factors for PEP. Repeated intubation attempts could increase anesthesia time for patients, enhance the risk of PEP and delay treatment selection (44). Furthermore, difficult cannulation can result in pancreatic duct visualization, spasms in the sphincter of Oddi and edema of the papilla, eading to pancreatic duct obstruction and pancreatitis (45). Generally, difficult cannulation is considered to be significant risk factor for PEP (45). To avoid repeated intubation attempts, advanced techniques, such as pancreatic duct wiring and pre-cut techniques could be used in clinical practice whenever possible to reduce the incidence of PEP (7). Diabetes, insulin resistance, obesity and hyperlipidemia were also reported to be independent risk factors for PEP (7,46,47). However, studies with larger sample sizes are required to verify these results. In addition, PEP could be promoted by injury to the pancreatic duct during papilla dilatation or by papilledema or spasm after dilation caused by ERCP (48).

In the present study, several risk factors, including age <60 years, cannulation time ≥10 min, SOD and pancreatitis history, showed statistically significant heterogeneity. The sources of heterogeneity were mainly clinical and methodological heterogeneity. Clinical heterogeneity was mainly due to differences between the inclusion and exclusion criteria in the studies such as country of participants, publication year and study type. Methodological heterogeneity was mainly due to the different types of studies included, such as prospective, retrospective and randomized controlled studies.

Andriulli *et al* (49) in 2002 and Chen *et al* (50) in 2014 also reported several risk factors for PEP. However, Chen *et al* (50) only searched three databases, namely MEDLINE, Elsevier and Springer links. In addition, the risk factors for PEP could also change with the development of instruments and technologies. Therefore, for the present study seven different databases were screened so that more potential risk factors could be identified based on studies that included >90 cases published between 2000 and 2022. Results from the present study do suggest that the risk factors leading to the PEP are worldwide because the patients included came from multiple countries, including China, Sweden, Japan, Korea, Greece, the USA and Germany. In particular, it was found that the risk of PEP in patients with ≥3 cannulation attempts was 8.98-fold higher compared with those with <3 cannulation attempts. In clinical practice, greater attention should be paid to this risk factor to avoid repeated intubation attempts.

However, the present study has some limitations. The quantity of the included studies was small. Therefore, the conclusions could not be sufficiently reliable. In addition, several eligible studies could not assess the value of risk factors, affecting the accuracy of the analysis. The risk factors summarized in the present study could also not be deemed as comprehensive due to the limited type of the included studies. Since patient- and technique/operation-related risk factors could be different among the different ethnicities and countries, further studies are needed to evaluate these differences.

In conclusion, PEP, the most common and serious condition caused by ERCP, is affected by several risk factors. Therefore, effective precautions should be taken as early as possible. Clinicians can prevent PEP from multiple perspectives, including patient selection, preoperative drug prevention and postoperative fluid resuscitation. Technique-related risk factors, such as the frequency and time of cannulation, are still the major risk factors for PEP in patients with CBDS. Therefore, clinicians should be skilled in the process of diagnosis and treatment to avoid damaging the surrounding tissues of the sphincter to reduce the difficulty of operation and reinjury, preventing PEP.

## Figures and Tables

**Table I:** Basic characteristics of the included studies.

First author/s, year	Publication country^a^	Study type	Sample size, n	Risk factor^b^	Newcastle-Ottawa Scale score	(Refs.)
Jia *et al*, 2017	China	Case-control study	92	2,5,6,9,12	6	(10)
Li, 2014	China	Case-control study	253	1,3,5,6,7	6	(11)
Liu *et al*, 2021	China	Case-control study	343	1,2,5,6,7,8,11	6	(12)
Wei *et al*, 2019	China	Case-control study	457	1,5,6	6	(13)
Ma *et al*, 2021	China	Case-control study	1,000	8,11,13,14	6	(14)
Zhang *et al*, 2022	China	Case-control study	986	5,7	7	(15)
Swahn *et al*, 2013	Sweden	Case-control study	12,718	1,4,7,9,10,13,14	8	(16)
Nakai *et al*, 2022	Japan	Prospective cohort study	800	3,6,9,10	7	(17)
Park *et al*, 2014	Korea	Case-control study	341	6,10,13	7	(18)
Saito *et al*, 2022	Japan	Retrospective cohort study	1,539	1,4,6,7,14	8	(19)
Christoforidis *et al*, 2002	Greece	Case-control study	513	1,2,5,6,9,10,14	6	(20)
Wang *et al*, 2009	China	Case-control study	2,691	1,4,9,10	6	(21)
Fujita *et al*, 2022	USA	Prospective cohort study	3,676	1,4,6,11,12,14	8	(22)
Meister *et al*, 2011	Germany	Retrospective cohort study	1,275	1,5,7,13	9	(23)
von Seth *et al*, 2015	Sweden	Prospective cohort study	666	1,9,10,14	9	(24)

^a^Publication country also represents the country of the study population.

^b^1, sphincterotomy of Oddi; 2, difficult cannulation; 3, cannulation attempts ≥3; 4, cannulation time ≥10 min; 5, history of pancreatitis; 6, pancreatic duct visualization; 7, gallstone; 8, history of sphincter of Oddi dysfunction; 9, age <60 years; 10, female; 11, guidewire entry of pancreatic duct; 12, para ampullary diverticulum; 13, bile duct stent placement; 14, endoscopic retrograde cholangiopancreatography time ≥45 min.

**Table II:** Meta-analysis results of risk factors for post-endoscopic retrograde cholangiopancreatography pancreatitis.

		Heterogeneity test			Overall effect test	
Risk factor	Study quantity, n	P-value	I^2^, %	Odd ratio (95% CI)	Z-value	P-value
Age <60 years	6	0.004	71.4	1.53 (1.06-2.21)	2.24	0.02
Female	6	0.617	0	1.42 (1.23-1.64)	4.77	<0.01
Sphincterotomy of Oddi	10	<0.01	89	1.56 (0.95-2.56)	1.76	0.08
Difficult cannulation	3	0.279	21.7	4.87 (2.73-8.68)	5.37	<0.01
Cannulation attempts ≥3	2	0.22	33.4	9.64 (4.16-22.35)	5.28	<0.01
Cannulation time ≥10 min	4	<0.01	80.9	2.37 (1.67-3.35)	4.85	<0.01
A history of pancreatitis	6	0.004	71.3	2.95 (1.58-5.51)	3.40	<0.01
Pancreatic duct visualization	9	0.007	62	3.63 (2.47-5.34)	6.55	<0.01
Gallstone	6	<0.01	92	1.87 (0.85-4.15)	1.55	0.12
Sphincter of Oddi dysfunction	2	0.046	74.8	5.72 (1.80-18.24)	2.95	0.003
Guidewire entry of pancreatic duct	3	<0.01	93	1.05 (0.25-4.40)	0.07	0.95
Para ampullary diverticulum	2	0.002	90	1.47 (0.34-6.44)	0.51	0.61
Bile duct stent placement	4	<0.01	93	2.39 (0.81-7.03)	1.58	0.11
Endoscopic retrograde cholangiopancreatography time ≥45 min	6	<0.01	91	1.55 (0.93-2.58)	1.66	0.10

All meta-analyses are performed using random-effects model. CI, confidence interval.

## Data Availability

The datasets used and/or analyzed during the current study are available from the corresponding author on reasonable request.
